# Long-term effect of antifungal therapy for the treatment of severe resistant asthma: an active comparator clinical trial 

**DOI:** 10.18502/cmm.5.4.1986

**Published:** 2019

**Authors:** Majid Mirsadraee, Sanaz Dehghan, Shadi Ghaffari, Niloofar Mirsadraee

**Affiliations:** 1 Internist and Pulmonologist, Professor, Islamic Azad University, Mashhad Branch, Mashhad, Iran; 2 Innovative Research Center, Faculty of Medicine, Islamic Azad University, Mashhad Branch, Mashhad, Iran; 3 MSc in Biology, Research Department, Kavosh High School, Mashhad, Iran; 4 MSc in Cell and Molecular Biology, Innovative Research Center, Faculty of Medicine, Islamic Azad University, Mashhad Branch, Mashhad, Iran

**Keywords:** Anti-fungal therapy, Asthma, Fungal sensitization, Itraconazole, Resistant asthma, Triazole

## Abstract

**Background and Purpose:**

Itraconazole therapy has been reported to control asthma in severe therapy-resistant asthma with fungal sensitization. The aim of this study was to investigate the impact of anti-fungal therapy on the treatment of severe asthma, irrespective of sensitization.

**Materials and Methods:**

This active comparator clinical trial was performed on 110 therapy-resistant asthmatic patients who were randomly assigned into two groups of case and control. The patients in the case group were administered 200 mg itraconazole twice a day and the control group received 10 mg prednisolone after breakfast for 4 months. The asthma control test (ACT) which was used as a marker for the global evaluation of treatment effectiveness (GETE) was applied as the primary endpoint parameter. Cough, dyspnea, and sleep disturbance were measured on a scale of 1-4, with 1 representing no symptom and 4 indicating severe exhausting disturbance.

**Results:**

Based on the obtained results, 71% of the itraconazole group demonstrated a marked improvement in the GETE score after a four-month treatment. Itraconazole was able to suppress clinical symptoms, including cough, dyspnea, and night symptoms, and their physical exam was indicative of normalization in 60% of the patients. On the other hand, the patients in the parallel group "prednisolone" were only able to control dyspnea. The ACT score represented a notable improvement with itraconazole (mean: 14 before the trial and >20 after the trial) and spirometry parameters underwent a considerable change from obstructive pattern to normal. Furthermore, adverse effects were only detected in 6% of itraconazole users.

**Conclusion:**

The results of this clinical trial indicted the effectiveness of antifungal therapy for the control of the clinical condition of a subgroup of patients with severe steroid-refractory asthma.

## Introduction

Severe form of asthma also known as treatment-resistance asthma which is poorly responsive or refractory to standard treatments affects less than 5% of patients [1]; nonetheless, it is responsible for 50% of the economic burden of asthma management [2]. Due to the ineffectiveness of available routine drugs with low side effects, the treatment of severe asthma poses a daunting challenge to lung specialists. Severe resistant asthma is highly unlikely to be linked to atopy and allergic conditions in other organs. Accordingly, attention has been turned to other conditions triggering treatment-resistance asthma by the exclusion of this large group of patients [3]. 

A new phenotype of asthma known as severe asthma with fungal sensitization (SAFS) has been introduced recently as a cause of severe asthma. This disease resembles allergic bronchopulmonary aspergillosis (ABPA) but without obvious bronchiectasis and high IgE titer [4]. More severe asthma results in more prevalent ABPA and SAFS (approaching 33% in severe asthma) [5]. This result contradicts the European espiratory society/American thoracic society (ERS/ATS) guidelines on severe/resistant asthma which did not suggest the evaluation and treatment of severe asthmatic subjects without evidence of ABPA [1]. This recommendation is apparently related to the fact that although antifungal therapy has the potential to treat ABPA [6], limited clinical trials have been performed on the effect of triazole therapy on SAFS [7] and the results of these studies have not been as consistent as ABPA [8]. Allergic responses to other fungi (i.e., allergic bronchopulmonary mycosis) highlight the importance of practical effectiveness of anti-fungal therapy in asthma. Therefore, experts need more data prior to restricting the administration of triazoles for the treatment of severe/resistant asthma requires [9]. 

On the other hand, diagnostic criteria for SAFS and many immature fungal diseases are more elusive in allergic diseases of the lung, in comparison with ABPA. Laboratory exams for sensitization to fungus are found to be ineffective and many patients with severe asthma, irrespective of their sensitization results, have benefited from anti-fungal therapy [7, 10]. In this respect, a clinical trial of anti-fungal therapy in severe/resistant asthma regardless of the SAFS diagnostic exam may contribute to the estimation of fungal disease burden and deepen our understanding of the benefits and adverse effects of triazole therapy on severe asthma, including SAFS and allergic broncho-pulmonary mycosis. With this background in mind, we designed a clinical trial the main inclusion criterion of which was severe/resistant asthma instead of positive testing for fungal disease (as used by previous studies for SAFS) [10].

The aim of this study was two-fold. Firstly, it attempts to identify the effectiveness of new triazoles in asthma treatment, and secondly to determine the possible adherence of anti-fungal treatment to the ERS/ATS guidelines. 

## Materials and Methods

*Study design*

**Figure1:**
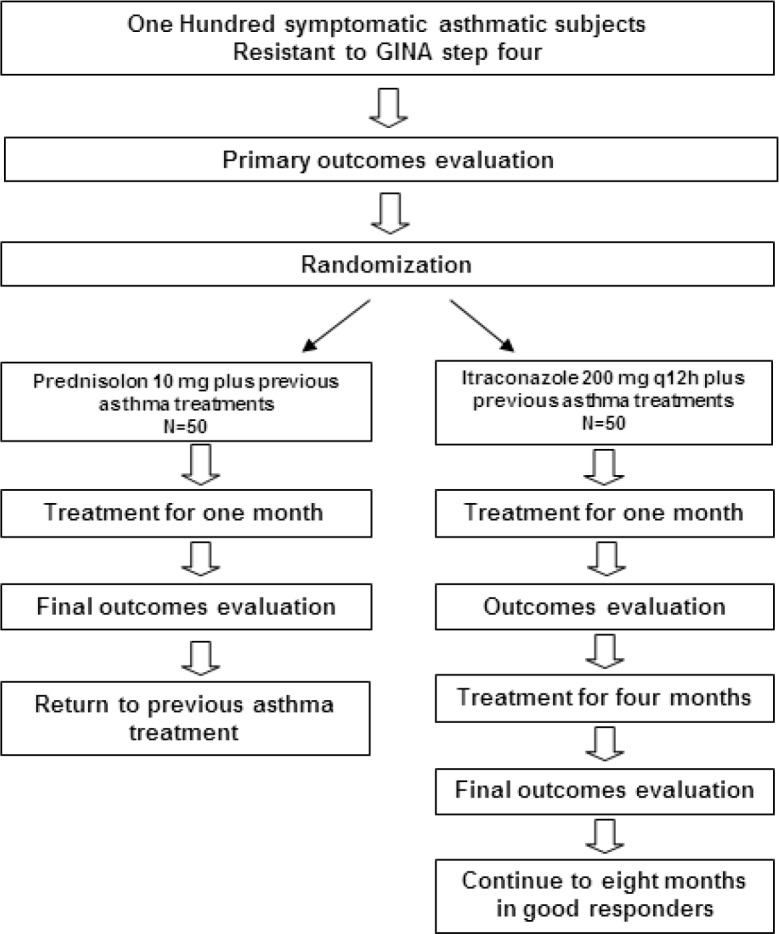
Study protocol in the clinical trial comparing itraconazole with prednisolone on asthmatic subjects resistant to the Global Initiative for Asthma(GINA) step 4 treatments

This study is a single blinded, active comparator, randomized, placebo controlled, clinical trial (Figure 1).

The current study was evaluated and approved by Institutional Review Board, our Local Ethical Committee (IR.IAU.MSHD.REC.1396.012) and our National Clinical Trial Registry (IRCT IRCT201410032695N7).

***Inclusion Criteria***

The present study was conducted on outpatients with asthma who were resistant to GINA stage 4 asthma treatments and required the initiation of treatment with prednisolone as stated in the GINA stage.

1. The age range of 15-70 years old, a documented history of uncontrolled asthma, and obtaining a minimum of three-month treatment with inhaled corticosteroids (ICS), long-acting beta 2 agonists (LABA), leukotriene modifiers with or without theophylline using the stepwise approach as defined by GINA strategy.

2. Forced expiratory volume in one second (FEV1) less than 80% predicted, apart from FEV1/FVC (Forced vital capacity) less than 75%.

3. Experience of either cough or dyspnea as confirmed by an investigator. 

4. No emergency situation for at least three days prior to the study. 

5. Already-treated associated rhino-sinusitis and/or gastro-esophageal reflux before the commencement of the study.

*Exclusion Criteria*

The main exclusion criteria entailed: 1) pregnancy, 2) breastfeeding, 3) elevated liver enzymes, 4) structural lung disease, and 5) systemic corticosteroid or omalizumab usage within 30 days prior to enrollment. It is worthy to note that computed tomography of the lung was used to evaluate the structure of the lung disease prior to enrollment.

*Antifungal treatment*

The participants were assigned into itraconazole and prednisolon groups based on computer-generated random table. The patients in the case group were administered an itraconazole capsule (purchased from Rouz Daru Company, Tehran, Iran or Tehran Daru Company, Tehran, Iran). The initial dose was specified as 200 mg twice a day for 120 days and then 100 mg twice a day for 120 days. On the other hand, the patients in the control group received a prednisolone tablet 10 mg after breakfast for one month. Previous drugs were continued. The subjects were examined after the course of one-month treatment and the medication was continued for the full four months course of therapy if it was tolerated and demonstrated satisfactory results.

*Primary and secondary outcomes*

The primary endpoint of this study was clinical improvement which was assessed using Asthma Control Test (ACT) questionnaire. At the same time, a range of clinical findings was evaluated as secondary outcomes, including cough frequency, dyspnea, sputum, chest tightness/discomfort, and auscultatory findings of the chest. In addition, the severity of clinical findings was examined using the verbal rating scale in the following way: cough and dyspnea (0)=none, 1 (mild)=less than two per week, 2 (moderate)=alternate date occurrence, 3 (severe)=daily non-continuous, 4 (very severe)=continuous day and night); sputum: 0=no sputum, 1=transparent, 2=white, 3= purulent (dark yellow or green). 

Improvements were assessed by the overall change of symptoms and were rated on a four-step verbal rating scale considering: (0) = the patient's status deteriorated, (1) =the symptoms remained unchanged since the first consultation, (2)=improvement of symptoms, but not complete disappearance of symptoms, (3)=complete improvement and resolution.

In addition, sleep quality was rated on a four-step verbal rating scale (0=sleep every night of the week, 1=sleep more than three nights per week, 2=sleep less than three nights per week, 3=unable to sleep in any of the nights of the week). Moreover, at each visit, body temperature was checked. 

Para-clinical evaluation entailed specific IgE against aspergillosis species, spirometry and fraction of expiratory nitric oxide (FENO). Spirometry was examined by Spirolab III( Mir company, Italy) and FENO test (Bedfont, England). Medication tolerance and side effects of itraconazole were evaluated separately and recorded up to one month after the treatment. Tolerance determination was carried out using the following scale: (0) = discontinuation of therapy due to adverse effects, (1) = moderate adverse effect occurs but does not require discontinuation of therapy, (2) = no evidence of adverse effects.

*Statistical analysis*

The study population included 50 patients in each case and control group. The sample size calculation was based on the mean change of the ACT score obtained from a previous pilot study. In addition, the normality of data was evaluated using the Kolmogorov-Smirnov test. 

Primary results and the frequency of side effects and discontinuation were determined by descriptive analysis and compared by Chi-square, Mann-Whitney U test, Kruskal Wallis, and student's t-test. Furthermore, the comparison of clinical and para-clinical findings between pre-therapy and post-therapy were made using McNemar, Wilcoxon signed-rank, and paired t-tests. 

## Results

The current study was carried out on a number of 101 subjects (51 participants in the itraconazole and 50 ones in the prednisolone group). Participants' mean age was reported as 52.29±15.42 years (Table 1). Female to male ratio was 3/1 and less than half of them lived in rural areas. The duration of disease previous to admission was reported to be between 5-456 months with an average of 99.2±90 months (8.4±7.9 years). Lung computed tomography was obtained as normal in 83% of the subjects and the others demonstrated some non-significant infiltrate, such as air trapping, scattered nodule, ground glass, or patchy small infiltrations. Moreover, all participants reported a chronic, stable course with little day-to-day variation, except for one subject with a prominent remittent course. All participants were administered a combinations of long-acting beta-agonists-inhaled corticosteroid(LABA-ICS) and anti-leukotrienes and more than half of the subjects (55.7%) consumed all five abovementioned drugs; however, the remaining patients demonstrated intolerance to one or more type of drugs (27.1% consumed four drugs, 11.4% three drugs and 5.7% two drugs). Furthermore, two-thirds of the participants reported other allergic symptoms (Table 1); nonetheless, 13% of participants (n=13) reported strong evidence of atopy. No significant differences were detected between itraconazole and prednisolone groups. 

Approximately 80% of subjects returned one month after the completion of treatment; however, 20-23% of them did not do so and were regarded as lost during the study (Table 2). Following one month, overall satisfaction and improvement were revealed only in the itraconazole group (Table 2). 

*Clinical findings*

All clinical findings, including night symptoms and wheezing evaluated by physical exam, demonstrated a significant improvement in the itraconazole group, as compared to prednisolone group (Table 3). A considerable number of subjects demonstrated constitutional symptoms which did not significantly change upon the administration of prednisolone; however, myalgia substantially decreased with itraconazole (Table 3).

**Table 1:** Demographic and basic data of resistant asthmatic subjects recruited in the clinical trial comparing itraconazole with prednisolone

	**Total**	**Itraconazole**	**Prednisolone**
**Number**	103	51	52
**Lost during study**		13 (25%)	14 (28%)
**Age (Years)**	52±15 (18-87)	49±15*	56±13
**Sex (Female/Male)**	76/23	39/12	39/12
**Duration of asthma (Years)**	8.4±7.9 (1-38)	8.3±7.8	8.7±8.1
**Residence (Urban/Rural)**	71/30	40/10	31/20
**Normal CT scan**	83%	80%*	100%
**Occupational air pollution**	12 (12%)	6 (12%)	6 (12%)
**Nasal allergy**	56 (56%)	31 (63%)	25 (52%)
**Urticaria**	52 (52%)	27 (52%)	25 (52%)
**Allergic conjunctivitis**	24 (24%)	13 (25%)	11 (22%)
**At least one allergic symptoms**	63 (63%)	35 (70%)	28 (54%)

*= Significant difference between itraconazole and prednisolone groups

**Table 2:** Clinical course of severe asthmatic subjects managed by prednisolone or itraconazole

	**Prednisolone**	**Itraconazole** **After 1 month**	**Itraconazole** **After 4 months**
**Become worse**	8 (20%)	1 (2.5%)	1 (3.6%)
**Get better but not complete**	22 (55%)	24 (58.5%)†	7 (25%)‡
**Complete feeling of healthy**	10 (25%)	16 (40%)†	20 (71.4%)‡
**Lost during study**	11 (22%)	10 (20%)	3 (2%)
**Needs to long term continue**	3 (6%)	-	24 (60%)
**Side effects-not discontinued**	6 (12%)	0 (0%)	0 (0%)
**Side effects-discontinued**	6 (12%)	2 (5.7%)	1 (4%)
**Well tolerance**	36 (76%)	33 (94.3%)	24 (96%)

†=Significant difference between case and control group after one month treatment with itraconazole

‡= Significant difference after the trial in the itraconazole group (paired t-test)

**Table 3:** Comparison of effects of itraconazole with the prednisolone on demographic and clinical findings of severe asthma

	**Total**	**Before trial**		**After one month**		**After 4 months**
		**Itraconazole**	**Prednisolone**	**Itraconazole**	**Prednisolone**	**Itraconazole**
**Cough **	92 (94%)	49 (96%)	43 (90%)	30 (59%)‡	38 (78%)	24 (48%)‡
**Dyspnea**	96 (96%)	50 (98%)	46 (94%)	31 (62%)†‡	48 (100%)	26 (52%)‡
**Sound on chest**	95 (95%)	49 (96%)	46 (94%)	26 (53%)‡	38 (78%)	22 (44%)‡
**Sputum**	44 (86%)	44 (86%)	33 (68%)	24 (47%)‡	36 (75%)	26 (50%)‡
**Chest pain**	62 (62%)	37 (77%)*	25 (52%)	16 (30%)‡	21 (42%)	17 (33%)‡
**Wheezing**	88 (89%)	45 (98%)	43 (90%)	15 (34%)†‡	43 (88%)	14 (30%)‡
**Night symptoms**	86 (86%)	43 (87%)	43 (89%)	15 (30%)†	28 (56%)	6 (12%)‡
**Fever**	22 (22%)	6 (12%)*	16 (32%)	4 (8%)	12 (23%)	2 (4%)
**Sweating**	61 (61%)	33 (65%)	28 (58%)	33 (65%)	38 (78%)	22 (44%)
**Myalgia**	84 (84%)	43 (84%)	41 (84%)	29 (57%)‡	32 (66%)	26 (52%)‡

AHR= Airway hyper-responsiveness, GERD= Gastero-esophageal reflux, PND= Post- nasal drip

*= Significant difference between case and control groups before the trial (chi square test)

†= Significant difference between case and the control groups after the trial (chi square test)

‡= Significant difference after the treatment with Itraconazole

(McNemar test)

In addition, the comparison of clinical staging indicated significant improvement in the mean scores of cough, dyspnea, and sleep following itraconazole treatment (Table 4). This was similarly the case for cough and dyspnea in the prednisolone group. Although the mean severity scores of cough and dyspnea in the itraconazole group were lower, as compared to the prednisolone group, they did not reach a significant level (Table 4). On the contrary, the use of itraconazole resulted in a significant reduction in sleep problems. Color and characteristics of sputum did not demonstrate a drastic change in the itraconazole and prednisolone groups (Table 4). Nonetheless, the ACT score increased considerably only in the itraconazole group indicating a significant difference between this group and the prednisolone group (Table 4).

*Physiological parameters*

All spirometry parameters except Forced expiratory volume in one second per Vital capacity (FEV1/VC) and Forced expiratory flow between 25-75% of vital capacity per FVC (EF_25-75_/FVC) presented a remarkable improvement following the treatment with itraconazole (Table 5). At the same time, the participants in prednisolone group were reported to improve in predicted FEF_25-75_ percent and FEF_25-75_/FVC. A comparison of the two groups revealed that all spirometry parameters were significantly higher in the itraconazole group. In addition, FENO test indicated a significant difference in neither group after the treatment. Mean blood eosinophil counts were mainly lower than 1000IU/ml of blood, and although it decreased after the treatment in the prednisolone group, this change was not statistically significant (Table 5). Mean serum IgE in all subjects at the commencement of the study was obtained as 465+/- 678 IU/ml (2.5-2500) and there was no significant difference after the trial in both groups (Table 5). 

***Effects of itraconazole after four months of therapy***

All clinical symptoms decreased significantly (Table 2) and more than half of the subjects did not demonstrate any sign of cough and dyspnea. In addition, sputum expectoration and night symptoms were not detected in about 80% of subjects (Table 3). The mean ACT score was obtained as more than 20 and the physical exams were normal in 70% of subjects (Table 2). Once more spirometry parameters demonstrated significant improvement and were reported to fall within the normal range (Table 4) and FENO decreased with a non-significant difference. Furthermore, blood cell count and serum IgE revealed non-significant changes (Table 4). 

***Global Evaluation of Treatment Effectiveness (GETE) or overall satisfaction and side effects***

Despite mild adverse effects associated with prednisolone, most patients demanded the medication discontinuation for reasons, such as cortonophobia and its possible side effects (Table 2). Overall satisfaction and feeling of symptoms resolution were significantly higher in the itraconazole group, as compared to the prednisolone group, although more than 80% of subjects in the prednisolone group noted partial resolution (Table 2). More than 70% of participants in the itraconazole group reported complete resolution of symptoms four months after the treatment. In addition, three subjects discontinued itraconazole due to its side effects and itraconazole was replaced with voriconazole.

**Table 4:** Comparisons of major clinical staging between prednisolone and itraconazole in participants with severe asthma

	**Total**	**Before trial**		**After one month**		**After 4 months**
		**Itraconazole**	**Prednisolone**	**Itraconazole**	**Prednisolone**	**Itraconazole**
**Cough**						
None	6 (6%)	2 (4%)	4 (10%)	14 (27%)	2 (10%)	13 (52%)
Mild	17 (17%)	9 (18%)	8 (16%)	8 (16%)	3 (15%)	8 (32%)
Moderate	19 (19%)	4 (8%)	15 (30%)*	6 (12%)	1 (5%)	2 (8%)
Severe	41 (41%)	30 (61%)	11 (22%)*	5 (10%)	3 (16%)	2 (8%)
Very severe	17 (17%)	6 (12%)	11 (22%)	1 (2%)	0 (0%)	0 (0%)
Mean	2.4±1.11	2.5±1.04‡	2.2±1.26	1.15±1.2‡	1.56±1.2¥	0.72±0.93‡
**Dyspnea**						
None	2 (2%)	1 (2%)	1 (5%)	13 (38%)	5 (27%)	12 (48%)
Mild	3 (3%)	3 (6%)	0 (0%)	11 (32%)	2 (11%)	7 (28%)
Moderate	16 (16%)	5 (10%)	11 (23%)	4 (12%)	6 (33%)	4 (16%)
Severe	67 (68%)	31 (61%)	37 (72%)	6 (18%)	4 (22%)	1 (4%)
Very severe	11 (11%)	11 (21%)	0 (0%)	0 (0%)	1 (2%)	1 (4%)
Mean	2.8±0.83	2.9±0.85‡	2.6±0.76	1.09±1.11‡	2.1±1.26¥	0.88±0.21‡
**Sputum**						
None	23 (23%)	7 (13%)	16 (32%)	35 (70%)	36 (75%)	40 (78%)‡
Translucent	2 (2%)	1 (2%)	1 (2%)	0 (0%)	0 (0%)	1 (2%)
White	30 (30%)	15 (30%)	15 (30%)	7 (13%)	8 (17%)	6 (12%)
Purulent	45 (45%)	28 (55%)	17 (36%)	9 (17%)	4 (8%)	4 (8%)
**Sleep**						
All night	40 (41%)	12 (24%)	28 (58%)*	23 (70%)†	(50%)	44 (88%)
>3 d /week	19 (19%)	11 (23%)	8 (16%)	7 (21%)	0 (0%)	4 (8%)
< 3 d/week	28 (29%)	20 (41%)	8 (16%)	3 (9%)†	(50%)	0 (0%)
None/week	11 (11%)	6 (12%)	5 (10%)	0 (0%)	0 (0%)	2 (4%)
Mean	1.23±1.05	1.4±0.99*	0.78±1.06	0.39±0.65†‡	1.2±1.06	0.2±0.34‡
ACT	14.16±2.83	13.12±4.37	14.1±3.22	19.8±4.05†‡	14±5.62	20.9±4.9‡

* Significant difference between case and control group before the trial

†=Significant difference between case and control group after the treatment with Itraconazole

‡= Significant difference after the trial in the Itraconazole group (paired t test)

¥= Significant difference after the trial in the placebo group (paired t test)

ACT= Asthma control test

**Table 5:** Comparison of overall asthma control test score, spirometry, and physiological evaluation of subjects enrolled in the trial for the treatment of severe asthma by itraconazole and prednisolone

	**Before trial**		**After one month**		**After 4 months**
	**Itraconazole**	**Prednisolone**	**Itraconazole**	**Prednisolone**	**Itraconazole**
**FVC (L)**	1.6±0.92	1.8±0.7	2.3±0.87†‡	1.69±0.68	3.1±1.84‡
**FVC percent**	55.2±22.23	60.3±16.65	71.8±18.8†‡	57.7±21.8	79±39‡
**FEV1 (L)**	1.3±0.73	1.14±0.45	1.9±0.8†‡	1.1±0.2	2.4±1.51‡
**FEV1 percent**	50.16±22.7	48.2±15.4	71.5±21.8†‡	47.8±17.9	82.5±30.4‡
**FEV1/FVC**	72.8±12.61	72.1±15.39	79.1±12.7†‡	64.7±10.3	89.5±0.7‡
**FEV1/VC**	70.82±22.7	78±20.3	76.6±14.6†	70±21.1	96.5±9.1‡
**FEF25-75 (L/S)**	1.21±0.8*	0.86±0.43	2.02±1.1†‡	0.9±0.59	2.6±1.95‡
**FEF25-75 percent**	36.8±21.83*	25.3±16.2	58.1±26.1†‡	33.2±28.4¥	75.5±40.3‡
**FEF25-75/FVC**	0.71±0.29*	0.51±0.3	0.83±0.28†	0.57±0.26¥	0.9±0.19
**FENO (PPM)**	36.8±29.2	28.6±25.2	34.6±26.5	35.2±22.1	29±17.9
**Lukocyte count**	9129±3378.8	9900±3093	8900±2524	9000±1414	8397±1596
**Eosinophile count**	446±699.9	703±676.1	682±773	180±28	1016±203
**Eosinophile percent**	5.7±7.11	10±12.5	8.1±9.4	2±0.4	5.1±7.5
**Neutrophile count**	5252±2993	6266±1824	5126±1754	4550±1343	3810±2354
**Neutrophile percent**	55.7±13.64*	63.7±3.3	57.6±11.2	50±7.07	45±24
**Serum IgE**	482±670	323±88	424±442	332±882	571±116

FVC= forced vital capacity; FEV1= Forced expiratory volume in one second; VC= vital capacity; FEF25-75= Forced expiratory flow between 25-75% of vital capacity; FENO= Fraction of expiratory nitric oxide; IgE= immunoglobulin E

*= Significant difference before and after the trial in the itraconazole group (paired t-test)

†=Significant difference between case and control group after a one-month treatment with itraconazole

‡= Significant difference after the trial in the Itraconazole group (paired t-test)

¥= Significant difference after the trial in the placebo group (paired t-test)

## Discussion

In this clinical trial, low dose prednisolone and itraconazole were administered to treat severe therapy-resistant asthma. Prior to trial, the subjects were treated by the step-wise approach as recommended by the GINA strategy and were symptomatic using a combination of 3-5 drugs. The results of the study were indicative of the effectiveness of both the prednisolone and itraconazole; nonetheless, the subjects' compliance with prednisolone was lower, as compared to most itraconazole-takers. In addition, the subjects who underwent treatment with itraconazole managed to continue the course of treatment and more than 70% of them were reported to be completely resolved (Table 2) and have a normal physical examination. Moreover, sound sleep was reported by more than 80% of the subjects (Table 3). The severity of asthma decreased in the remaining subjects, and severe symptoms persisted in a very small percentage of participants (Table 4). Furthermore, spirometry results suggested complete improvement after a four-month treatment with itraconazole which was not the case with the administration of prednisolone and many other drugs used for the treatment of severe therapy-resistant asthma [4,13; Table 5). FENO decreased in the itraconazole group; however, the difference was not significant which can be attributed to the accuracy of the measurement device. Moreover, no marked change was detected in serum IgE and the blood inflammatory cells, including eosinophil (Table 5). 

Omalizumab is a safe treatment with 80% effectiveness as measured by the global evaluation of treatment effectiveness (GETE)[11]. Korn et al.[12] reported excellent or good results in more than 80% of the patients (70% in the present study). FEV1 revealed a significant improvement in treatment with omalizumab, as compared to the control basic asthma treatment group, although it did not become completely normal as in the present study with itraconazole. Nonetheless, FEV1 and FEF_25-75 _did not change significantly after omalizumab therapy in two other studies conducted in Japan [13]. To make matters worse, serious adverse events, such as aggravated asthma^, ^were observed in 16.5% of omalizumab-treated patients [12, 15]. In another study, itraconazole was also used as an aid to omalizumab therapy to reduce the level of IgE [14]. Therefore, it can be concluded that itraconazole exerted a more pronounced effect on asthma, as compared to omalizumab which is pertinent to the impact on the source of IgE production rather than making better non- specific control of asthma.

Association of severe asthma with mold sensitivity was confirmed by skin tests and the radio-allergosorbent test (RAST) in which mold sensitivity was more prevalent in severe asthma than mild to moderate asthma [15, 16]. However, taking into account the important role of antifungal agents in the treatment of asthma led us to believe that the growth of molds *in situ* is responsible for mold stimulation (whether colonize or invaded), rather than airborne mold allergen exposure. In this condition, the course of asthma could be continuous and therapy-resistant. 

New triazoles were used in ABPA; nonetheless, clinical trials for SAFS are limited and only five clinical trials conducted on a total of 160 subjects have been published [7, 10, 24, 25, 26]. The results of a study conducted by Pasqualotto et al. [7] were suggestive of the positive impacts of itraconazole on SAFS concerning the reduction of eosinophilic inflammation and improvement of lung function. 

The Fungal Asthma Sensitization Trial (FAST) study [10] is the best multicenter placebo-controlled clinical trial on SAFS treated by itraconazole. In the FAST study, side effects were observed in a considerable number of patients; however, treatment modification was needed in a limited number of subjects. In the present study, three subjects (6%) demonstrated severe side effects of itraconazole which required discontinuation of therapy. 

The most disappointing study on the effects of the new triazole on SAFS is the EVITA 3 study [17] that revealed no difference between the voriconazole and placebo groups in the rate of exacerbation and the Asthma Quality of Life questionnaire score. Nonetheless, there were some major problems in the method of this study which make us accept the results of this study with caution since any of these factors is enough to influence the results of the study. These factors included a low sample size, smoking in 40% of subjects, bronchiectasis in half of the subjects, and using prednisolone in 28-32% of them.

Accordingly, systemic therapies with new triazoles are effective in severe asthma; however, nebulized amphotericin was ineffective in the treatment of SAFS subjects [18] which may indicate a non-bronchial surface origin of aspergillosis or resistant strains to amphotericin. Nonetheless, according to the female preponderance revealed in the current study and previous report [7], it is assumed that vaginal colonization may be a potential source for fungal growth; therefore, local antifungal treatment may help to better treat and eradicate fungal burden. *[*4*, *19-21*].*

Increasing prevalence of SAFS is of great concern to the authors of the current article. Increased use of antibiotics, inhaled or oral corticosteroids, and more in-door humid weather provided by new air-conditioning systems have been proposed as the leading causes of this high prevalence. Inhaled corticosteroids are indicated to increase serum IgE in ABPA patients [22]; therefore, they may predispose the milieu of bronchial surface fungi growth. Atopic allergic asthma is believed to be most effectively treated with ICS, whereas the most resistant and severe asthma is the best candidate for triazole therapy.

## Conclusion

In severe asthma, in cases of resistant to most of treatments, antifungal therapy is very effective for the treatment of severe steroid-refractory asthma.

## Author’s contribution

M.M. designed the study, collected and analyzed the data, and wrote the manuscript. S.D. contributed to study design, data collections, and statistical analysis. Sh.Gh. collected and analyzed the data. N. N. analyzed the data and wrote the manuscript.

## Conflicts of interest

The authors declare that they have no conflict regarding the publication of this article. 

## Financial disclosure

The current study was supervised by Research Deputy of Islamic Azad University, Mashhad branch. No financial support was accepted from drug companies or other institutes in this study.
